# Why Do Parents Prefer to Know the Fetal Sex as Part of Invasive Prenatal Testing?

**DOI:** 10.5402/2012/524537

**Published:** 2012-12-12

**Authors:** Angelique J. A. Kooper, Jacqueline J. P. M. Pieters, Alex J. Eggink, Ton B. Feuth, Ilse Feenstra, Lia D. E. Wijnberger, Robbert J. P. Rijnders, Rik W. P. Quartero, Peter F. Boekkooi, John M. G. van Vugt, Arie P. T. Smits

**Affiliations:** ^1^Department of Human Genetics, Nijmegen Medical Centre, Radboud University Nijmegen, 6500 HB Nijmegen, The Netherlands; ^2^Department of Obstetrics and Gynaecology, Nijmegen Medical Centre, Radboud University Nijmegen, 6500 HB Nijmegen, The Netherlands; ^3^Department of Obstetrics and Gynaecology, Erasmus MC, University Medical Centre Rotterdam, 3015 GE Rotterdam, The Netherlands; ^4^Department of Epidemiology, Biostatistics and HTA, Nijmegen Medical Centre, Radboud University Nijmegen, 6500 HB Nijmegen, The Netherlands; ^5^Obstetrics and Gynaecology, Rijnstate Hospital, 6815 AD Arnhem, The Netherlands; ^6^Obstetrics and Gynaecology, Jeroen Bosch Hospital, 5200 ME ‘s-Hertogenbosch, The Netherlands; ^7^Obstetrics and Gynaecology, Medical Spectrum Twente, 7500 KA Enschede, The Netherlands; ^8^Obstetrics and Gynaecology, St. Elisabeth Hospital, 5022 GC Tilburg, The Netherlands

## Abstract

*Objectives*. The aim of this study was to determine whether prospective parents, primarily referred for prenatal diagnosis to exclude Down syndrome, prefer to know the fetal sex as part of invasive testing. *Methods*. In this prospective study 400 pregnant women undergoing amniocentesis were invited to answer a questionnaire, including information about demographic factors, current pregnancy, and previous children. In two open-ended questions they were asked why they wanted to know the fetal sex after amniocentesis or ultrasound investigation. Scores were given for reasons that could have played a role in the wish whether or not to know the sex of their unborn child. *Results*. A total of 210 (52.5%) questionnaires were completed. Overall, 69.0% was interested to know the fetal sex as part of the diagnostic test result. The most important reasons were curiosity (77.8%), “just want to know” (68.0%), and “because it is possible” (66.8%). The overall knowledge of sex chromosomal disorders appeared low and did not seem to affect the parent's wish to know the fetal sex. Almost all women (96.6%) planned to have a 20-week ultrasound scan and 96.2% thought the scan to be reliable in detecting the fetal sex. A minority (28%) was willing to learn the fetal sex by ultrasound examination, whereas 65% preferred to learn the fetal sex only after the amniocentesis. *Conclusion*. Personal values affect the parental desire to know or not to know the fetal sex. This does not appear to be affected by invasive prenatal testing and/or genetic knowledge of sex chromosomal disorders.

## 1. Background

For prospective parents the option of knowing the fetal sex forms an important aspect during pregnancy. Sometimes early determination of fetal sex is clinically indicated, for example, for women who are carrier of an X-linked genetic condition and for who male foetuses are primarily at risk. In these cases the fetal sex is determined in free fetal DNA isolated from maternal plasma early during gestation (8 weeks; [1–3]). For most other couples, however, knowing the fetal sex before birth is only possible after ultrasound examination or prenatal invasive testing by chorionic villi sampling or amniocentesis. In most prenatal centres, parents are asked whether they want to have the sex of the fetus reported before they undergo an invasive procedure using traditional karyotyping or rapid aneuploidy testing. With traditional karyotyping it is obvious that sex chromosomes are an integral part of the test result. Sex chromosomal disorders such as Turner- (45,X), Klinefelter- (47,XXY), Triple X- (47,XXX), and XYY-syndrome are a part of the diagnostic karyotyping tool. However, with rapid aneuploidy testing it is possible to in- or exclude sex chromosomal markers. Sex chromosomal aneuploidies (SCAs) in routine prenatal invasive testing are often incidental and present unforeseen findings to the parents, whereas their diagnostic significance is uncertain [4].

In The Netherlands, as in most other Western European countries, second trimester ultrasound screening for fetal anomalies (20-week scan) has become a standard part of prenatal care [5–7]. The accuracy of sex determination by ultrasound examination at 18–20 weeks ranges between 92% and 100% [8–10]. Herewith, the prenatal determination of fetal sex can be carried out noninvasively upon parental request.

 In this prospective study we assessed the factors associated with parents' preferences to know or not to know the fetal sex after amniocentesis and whether knowledge of sex chromosomal disorders influences the preference to know the fetal sex after amniocentesis or ultrasound examination. 

## 2. Methods

From May 2009 through May 2010, a questionnaire was offered to 400 women at the time of their amniocentesis in the hospitals participating in the Network Prenatal Diagnosis Nijmegen (NPDN): Radboud University Nijmegen Medical Centre, Rijnstate Hospital Arnhem, St. Elisabeth Hospital and TweeSteden Hospital Tilburg, Medical Spectrum Twente Enschede, and Jeroen Bosch Hospital, ‘s-Hertogenbosch. The reason for referral was advanced maternal age or increased risk after first trimester screening. The questionnaires were handed out to the women attending amniocentesis. The anonymous questionnaire was to be completed before the procedure was initiated or was taken home and filled in afterwards. The questionnaire used was adapted from a previous study by Bauman et al. [11] and consisted of multiple choice, open-ended, and five-point scale questions. 

The multiple-choice questions concerned sociodemographic variables (nationality, religion, marital status, and educational level) for the participant and her partner, obstetric variables (whether the pregnancy was planned and intended), and the method of reproduction. Furthermore the participant and her partner were asked whether they

1. opt for a 20-week scan,
2. think the 20-week scan is reliable in fetal sex determination,
3. have a preference for a particular sex,
4. have the desire to know the fetal sex,
5. have preference to know the fetal sex after amniocentesis or after the 20-week scan,
6. have previous children and whether the participant knew the sex of any of them before birth/prenatally,
7. have made up a name for their unborn child,
8. have the desire to have more children,
9. have people in their neighbourhood who know the sex of their unborn child,
10. are influenced by people to know or not know the fetal sex,
11. are familiar with sex chromosomal aberrations,
12. move or rebuild their house depending on the sex of the foetus,
13. tell the fetal sex to others.

The questionnaire included two open-ended questions about the reason why parents want to learn the fetal sex after amniocentesis or ultrasound, respectively. In the five-point numeric scale questions, scores had to be given for reasons that may have played a role in the parents' decision whether or not to discover the sex of their unborn child. 

The three-point numeric scale questions examining more in detail why wanting to know the fetal sex concerned

(14) just want to know the fetal sex,
(15) curiosity,
(16) impatience,
(17) sex preference,
(18) naming the child,
(19) preparation/planning,
(20) emotional attachment with the unborn child,
(21) prepare older siblings,
(22) shopping,
(23) sex preference partner,
(24) want no surprises,
(25) because it is possible.

The three-point numeric scale questions examining more in detail why not wanting to know the fetal sex concerned

(26) just do not want to know,
(27) surprise at birth,
(28) no sex preference,
(29) it is more fun not knowing,
(30) my partner does not want to know.

The collection of data was carried out till November 2010. Analysis of data was performed using SPSS for Windows (SPSS Inc., Chicago, IL, USA). Basic descriptive statistics were used. The influence of multiple factors on maternal desire to know the fetal sex was tested using the Chi square or Fisher exact test; differences between maternal and paternal/partner desire to know the fetal sex and sex preference were calculated based on the McNemar test. Statistical significance was set at *P* < 0.05.

## 3. Results 

The questionnaire was completed by 210 (52.5%) women. Overall, 94.8% were married or cohabiting couples. The ethnic background of the expectant mother was mainly Dutch (93.8%), 57.1% of the participants were nonreligious. Just over half of the expectant mothers (51.2%) followed higher professional education or university. In 81.0% of the couples the pregnancy was planned, in 89.1% the pregnancy occurred by spontaneous conception, and only one pregnancy was unintended. Demographic variables, pregnancy information and knowledge are shown in Table 1.

### 3.1. Finding out the Sex of the Baby

Overall, 69.0% of pregnant women and 77.2% of the partners were interested to know the fetal sex as a result of their amniocentesis (*P* = 0.02, McNemar). For 31.4% of the participants this was their first pregnancy, and 68.6% had previous children. Having one or more children did increase the percentage of expectant mothers wanting to know the sex of the fetus from 62% to 72%, although this increase was not significant (*P* = 0.18, Chi square test). Further, 44.6% of the women with children found out the sex of all their previously born children before birth, 10.1% knew in a part of their pregnancies, and 45.3% did not know the sex. After this pregnancy the family would be completed in 60.3% of the participants. This resulted in a single-child family in 9.5%, a two-child family in 54.0% and a desire for three children in 28.6%. Mothers wishing additional children in the future wanted to learn the sex of the baby less often (52.0%) as compared to mothers who expected to complete their family after the current pregnancy (75.4%) (*P* = 0.02, Chi square test).

Of the expectant mothers, 69.9% had thought of a name for their unborn child. The majority thought of names for both sexes (62.7%). When the expectant mother had only been thinking about one name, slightly more mothers had only thought of a boy's name (4.3%) rather than a girl's name (2.9%). Having already picked out a baby's name is not associated with maternal desire to know fetal sex (*P* = 0.16, Chi square test). None of the participants was planning to move or rebuild their house based on the sex of the baby. An overview of data about pregnancy history and childbirth plans is shown in Table 2. 

In 60.7% the expectant mother who wanted to know the sex of the baby was planning to tell this to family and friends. Almost 80% of them knew people in their environment who wanted to know the sex of their unborn child. In 97.2% the participants stated not to be influenced by others to know the fetal sex. There was no significant association between the level of maternal education and the expectant mothers desire to find out the sex of the baby (*P* = 0.34 Fisher Exact).

### 3.2. Parental Sex Preference

Only 13.8% of the expectant mothers and 18.5% of the partners did have a strong fetal sex preference, respectively (*P* = 0.15, McNemar). In 3.8% of the mothers and 11.6% of the partners there was preference for a boy (*P* = 0.004, McNemar), and in 10% and 6.9%, respectively, preference for a girl (*P* = 0.12, McNemar). Data of maternal and partner sex preferences and desires to know the fetal sex are shown in Table 3. Maternal sex preference did not differ between expectant mothers with or without previous children (*P* = 1.00, Fisher Exact). A higher preference for a son (18.6%) was noticeable when the expectant father/partner had one daughter and no sons; a higher preference for a daughter (12.0%) was noticeable when the expectant mother had one son and no daughters.

### 3.3. Knowledge of the 20-Week Scan and Sex Chromosomal Abnormalities

96.6% of the expectant mothers obtained a 20-week scan; 96.2% thought this scan is reliable in detecting the fetal sex. The question on having any knowledge of sex chromosomal aberrations (SCAs) was answered positive in 12 (7.7%) of the respondents. Examples of such aberrations were given for Turner syndrome (*N* = 2), Klinefelter syndrome (*N* = 1), Triple XXX (*N* = 1), XYY (*N* = 1), and Turner and Klinefelter syndrome (*N* = 2). Also positive answers without any specific example (*N* = 5) were given. The educational level (lower/middle education level versus higher education level) was significantly associated (*P* = 0.01, Fisher Exact) with knowledge of SCA: of all women with knowledge of SCA 83.3% completed higher professional education or university. Knowledge of SCAs did not influence the maternal preference to know the fetal sex (*P* = 0.22, Fisher Exact). 

### 3.4. Reasons for Wanting to Know or Not Know the Fetal Sex

**Figure 1:**
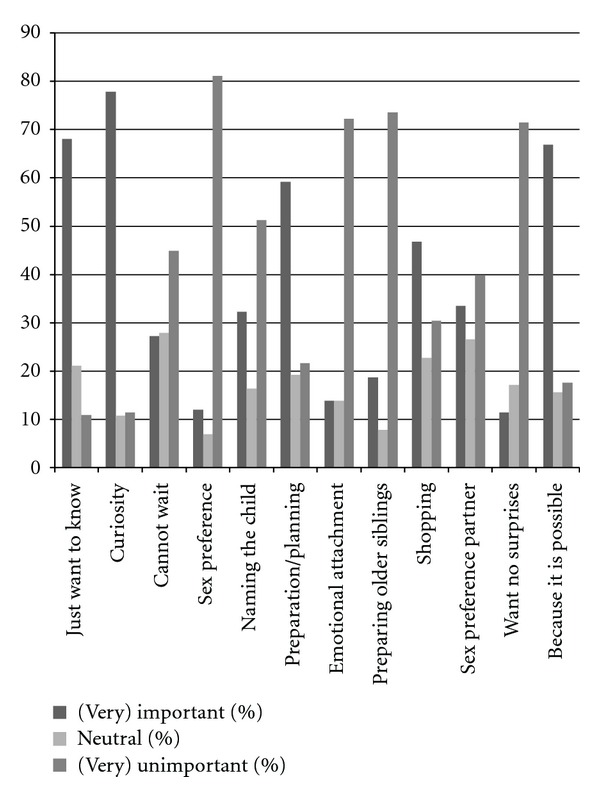
Reasons for wanting to know the fetal sex. The reasons that scored the highest percentage for being (very) important were curiosity (77.8%), “just want to know” (68.0%), and “because it is possible” (66.8%). Most (very) unimportant factors for wanting to know the fetal sex were the aspect of sex preference (81.1%), for preparing older siblings (73.5%) and for emotional attachment to the child (72.2%).

**Figure 2:**
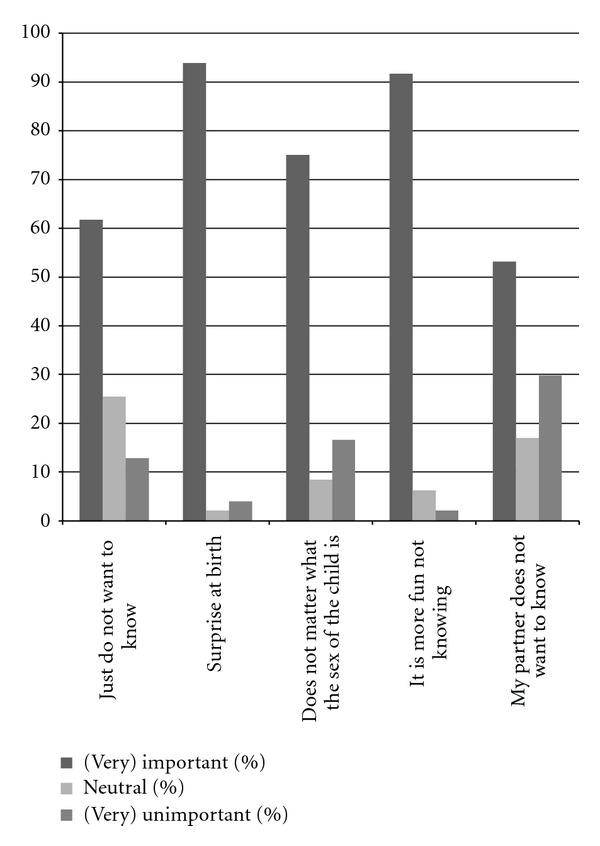
Reasons for not wanting to know the fetal sex. The two reasons that showed the highest percentage in being important for not wanting to know the fetal sex were “surprise at birth” (93.9%) and “it is more fun not knowing” (91.7%).

Reasons for wanting to know the fetal sex are shown in Figure 1. The reasons that scored the highest percentage for being (very) important were curiosity (77.8%), “just want to know” (68.0%) and “because it is possible” (66.8%). Most (very) unimportant factors for wanting to know the fetal sex were the aspect of sex preference (81.1%), for preparing older siblings (73.5%) and for emotional attachment to the child (72.2%).  Figure 2 shows the reasons for not wanting to know. The two reasons that showed the highest percentage in being important for not wanting to know the fetal sex were “surprise at birth” (93.9%) and “it is more fun not knowing” (91.7%). 

### 3.5. Parents' Willingness to Detect the Fetal Sex by Ultrasound

**Figure 3:**
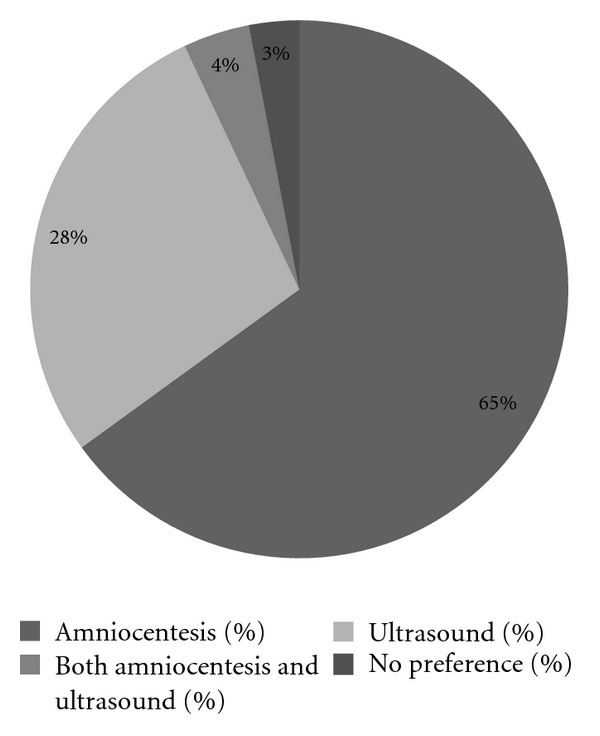
A minority of 28% of the responders were willing to be informed about the fetal gender at the time of the 20-week ultrasound examination, 65% preferred this information to be included in the amniocentesis result, 4% after both tests, and 3% was without preference for either types of test.

A minority of 28% of the responders were willing to be informed about the fetal gender at the time of the 20-week ultrasound examination, 65% preferred this information to be included in the amniocentesis result, 4% after both tests, and 3% was without preference for either types of test (Figure 3). In total 130 (61.9%) women added comments in the first open-ended question why they wanted to know the fetal sex after amniocentesis, and 61 (29.0%) responded to the second open-ended question why they wanted to know the fetal sex after ultrasound scan. For both open-ended questions four major considerations emerged from the analysis of the responses: because I have a diagnostic test, certainty, practical reasons, and the risk of the invasive test.

#### 3.5.1. Because I Have a Diagnostic Test

Most women (*N* = 45, 34.6%) expressed the wish to know the fetal sex after their amniocentesis because they undergo the invasive procedure. One of these women reported “*The amniocentesis is done for another reason, the pleasant thing of testing is the possibility to know the sex of your baby*.”

#### 3.5.2. Certainty

Most women (*N* = 37, 28.5%) expressed to know the fetal sex after their amniocentesis because this will be more reliable than knowing the fetal sex after ultrasound examination: “*Because you have a 100% certainty*” and “*It is not always possible to detect the fetal sex by ultrasound*.” 

#### 3.5.3. Practical Reasons

Twenty-two (16.9%) women wrote comments indicating that they wanted to know the sex of their baby after amniocentesis because of practical reasons. The same reason was expressed in the group who wanted to know the fetal sex after the ultrasound scan in 20% of the responders: “*When I know the sex of the baby I feel confident about baby shopping*” and “*It is practical for decorating the baby's room*.” 

#### 3.5.4. Risk of the Invasive Test

The noninvasive character of fetal sex determination by ultrasound was positively expressed in 16% of the women. Women curious to know the fetal sex did not want to take the risk of a miscarriage if invasive testing is not indicated for medical reasons: “*Knowing the sex of the baby is less important. When you know the fetal sex by ultrasound you do not take a risk to lose the pregnancy*.”

## 4. Discussion 

This study focused on pregnancies at risk for Down syndrome and was aimed at assessing why parents prefer to know the fetal sex after invasive prenatal testing. Previous studies showed percentages ranging from 58% to 64% among respondents for wanting to know the sex of their baby after ultrasound examination without undergoing an invasive procedure [11, 12]. In our study, 69.0% of the mothers and 77.2% of the partners were interested to know the fetal sex after their amniocentesis. Shipp et al. reported that the percentage of wanting to know the fetal sex by ultrasound did not differ between first, second, and third trimester [12]. Our higher percentage of desire to know may be explained by the fact that we studied a different population of pregnant women, that is, it included pregnant women with a medical reason for invasive testing and of which 68.6% had previous children. Although not significant, our study showed an increase of expectant mothers wanting to know the sex of the fetus and having one or more children. In western societies fetal sex preference is mostly based on social and psychological reasons [13]. In The Netherlands, prospective parents usually do not express their preference for a male or female child [14]. In conformity with the report of Jacobsen et al. [15] we found a slight parental preference to have at least one child of his or her own sex.

 The main reasons for parental desire to know the fetal sex were curiosity, “just want to know,” and “because it is possible.” The first two outcomes were also found in previous studies from Bauman et al. and Shipp et al. [11, 12]. Not wanting to know in order to have a surprise at birth was also the most important reason in both of these studies. 

 Our study showed that 28% of the prospective parents wish to know the sex of their baby after ultrasound examination. In the second and third trimester, fetal sex determination is based on direct visualization of the external genitalia of both males and females. Almost all women in our study population planned to have a 20-week scan and they expected the ultrasound scan to be reliable in detecting the fetal sex. Although assessed by one question only, the overall knowledge on sex chromosomal aberrations (SCAs) appeared to be poor. This may be due to the fact that the amniocentesis was carried out primarily to exclude Down syndrome. Although in our prenatal clinic pretest counseling includes sex chromosome aneuploidies, names of the involved syndromes are not specifically mentioned. This might explain the low percentage of respondents (7.7%) that could actually name an example of an SCA, but could also be viewed as an overall lack of knowledge on SCAs and the subsequent clinical consequences. It is worth noting that knowledge on SCA did not influence the preference to know the fetal sex after amniocentesis or ultrasound examination. So, it appears that parental desire to know or not know the fetal sex as a part of routine invasive prenatal testing for Down syndrome is in general not affected by genetic knowledge of sex chromosomal disorders. This study contributes to the discussion whether prenatal diagnostic testing to exclude Down syndrome should include determination of the fetal sex. Taking into account that pregnant women are primarily tested to exclude Down syndrome, the diagnosis of an SCA may prevail moral and ethical dilemmas to continue or terminate the pregnancy in case an SCA is detected [16, 17]. Replacement of traditional karyotyping by rapid aneuploidy testing for chromosomes 13, 18, and 21 only would avoid these incidental SCA findings. 

 With molecular rapid aneuploidy testing, parents could be given the autonomy to choose for diagnostic testing with or without inclusion of sex chromosomal markers. By doing so, they will be made aware of the fact that fetal sex determination may result in SCA detection. From a professional's point of view early SCA detection may provide opportunities for early support and treatment of certain health and developmental problems and, as such, may represent an advantageous step towards future healthcare for the child [18].

Personal values appear to affect parental desire to know or not to know the fetal sex. Apparently, this is not influenced by routine invasive prenatal testing and seems generally not influenced by genetic knowledge of sex chromosomal disorders. Although parents are aware that the fetal sex can be determined with high accuracy by ultrasound examination, most parents prefer to know the fetal sex based on an invasive diagnostic test result. More research is needed to assess the parental knowledge about SCA in more detail and to evaluate the pretest counselling content and the effect of knowledge about SCA on genetic testing. This aspect will probably become more important because of the upcoming implementation of noninvasive prenatal diagnosis (NIPD). Herewith early fetal sex determination using cell-free fetal DNA in maternal plasma will probably become a part of routine prenatal diagnosis.

## 5. Limitations

A limitation of the study is the premise that the questionnaire was filled in by the pregnant woman together with her partner while waiting for their amniocentesis. It is also possible that the questionnaire was taken home and filled in afterwards by the expectant mother alone. To make a conclusion about the paternal/partner's desire to know the fetal sex, a separate questionnaire should be assessed.

**Current Knowledge on This Topic**

- parents feel strongly about whether or not to learn the sex of their fetus;
- parents' desire to know the fetal sex during a prenatal ultrasound are families in which the pregnancy was unplanned, those in which fetal sex would influence living arrangements or future childbearing plans, and those of lower socioeconomic status.

**What This Study Adds Is as Follows**

- Personal values affect the parental desire to know or not to know the fetal sex as a part of invasive prenatal testing.
- Parental desire to know or not to know the fetal sex does not appear to be affected by invasive prenatal testing and/or genetic knowledge of sex chromosomal disorders.

## Figures and Tables

**Table 1:** Demographic variables, pregnancy information, and knowledge.

Variable (*N* = numbers of respondents)	*N*	Percentage
Nationality mother (*N* = 210)		
Dutch	197	93.8
Non-Dutch	13	6.2
Religious (*N* = 210)		
Yes	90	42.9
No	120	57.1
Marital status (*N* = 210)		
Married	99	47.1
Cohabitant	100	47.7
Single	3	1.4
Living apart together	8	3.8
Education mother (*N* = 209)		
Low secondary education	23	11.0
Middle secondary education	12	5.7
High secondary education	5	2.4
Middle professional education	62	29.7
High professional education	68	32.5
University	39	18.7
Education partner (*N* = 202)		
Primary school	3	1.5
Low secondary education	23	11.4
Middle secondary education	9	4.5
High secondary education	7	3.5
Low professional education	1	0.5
Middle professional education	72	35.6
High professional education	51	25.2
University	36	17.8
Reproduction method (*N* = 183)		
Spontaneous	163	89.1
Assisted reproduction	20	10.9
Will have a 20-week scan (*N* = 210)		
No	2	1.0
Yes	203	96.6
Uncertain	5	2.4
Thinks the 20-week scan is reliable in fetal sex determination (*N* = 209)		
No	8	3.8
Yes	7	37.3
Yes, very reliable	123	58.9
Do you know sex chromosomal aberrations? (*N* = 155)		
Yes	12	7.7
No	143	92.3

**Table 2:** Pregnancy history and childbirth plans.

Variable (*N* = numbers of respondents)	*N*	Percentage
Number of previous children (*N* = 210)		
None	66	31.4
1	95	45.2
2	37	17.6
3	10	4.8
4	1	0.5
5	0	0
6	1	0.5
Did you know the fetal sex in previous pregnancies? (*N* = 148)		
Yes, in all pregnancies	66	44.6
Yes, but not in all pregnancies	15	10.1
No	67	45.3
Do you want to have more children? (*N* = 209)		
Yes	25	11.9
No	126	60.3
Don't know yet	57	27.3
Depends on the sex of this baby	1	0.5
Have you already picked out your baby names? (*N* = 209)		
Yes, a name for a girl	6	2.9
Yes, a name for a boy	9	4.3
Yes, a name for both sexes	131	62.7
No	63	30.1
Are you going to tell others the sex of your unborn child? (*N* = 163)		
No	64	39.3
Yes, to my family	34	20.9
Yes, to my friends	1	0.5
Yes, to my family and friends	64	39.3
Are there people in your environment who know the sex of their unborn child? (*N* = 209)		
No	20	9.6
Yes, everybody I know	16	7.7
Some people I know	151	72.2
I do not know	22	10.5
Are you influenced by people in knowing the sex of your unborn child? (*N* = 208)		
No	202	97.2
Yes, because of them I want to know	3	1.4
Yes, because of them I don't want to know	3	1.4

**Table 3:** Maternal and paternal/partner sex preference and desire to know the fetal sex.

Variable (*N* = numbers of respondents)	*N* (%) pregnant woman	*N* (%) partner
Wants to know the fetal sex (*N* = 210 and *N* = 184)		
Yes	145 (69.0)	142 (77.2)
No	65 (31.0)	42 (22.8)
Fetal sex preference (*N* = 210 and *N* = 189)		
Yes, boy	8 (3.8)	22 (11.6)
Yes, girl	21 (10.0)	13 (6.9)
No	181 (86.2)	154 (81.5)
